# Enhancing cervical cancer knowledge among women of reproductive age: a dialogue-based community health education intervention in rural Kisumu County, Kenya

**DOI:** 10.1186/s12905-024-03075-2

**Published:** 2024-06-06

**Authors:** Ochomo Edwin Onyango, David Masinde, Collins Ouma

**Affiliations:** School of Public Health and Community Development, Maseno University, Private Bag, Maseno, Kenya

**Keywords:** Cervical cancer, Community health, Education, Dialogue, Women of reproductive age

## Abstract

**Background:**

Cervical cancer is a leading cause of cancer death among women of reproductive age despite being treatable if it is diagnosed early. Early diagnosis is possible through regular screening through the public health system. However, screening rates remain low in many low- and middle-income countries, including Kenya, where the screening rate currently stands at 16–18%. The low screening rates are attributed to, among other factors, low knowledge about cervical cancer and the available screening options among women of reproductive age. The current study evaluated the effectiveness of dialogue-based community health education by trained community health volunteers (CHVs) in improving cervical cancer knowledge among women of reproductive age (WRA) in rural Kisumu County.

**Methods:**

This was a longitudinal pre- and post-intervention study with a control group. The knowledge of women of reproductive age was assessed at baseline in both the intervention and control groups, followed by dialogue-based community health education in the intervention arm. A final end-line knowledge assessment was performed. The scores at baseline and at the end of the study were compared to assess changes in knowledge due to the intervention. The proportion of WRA with improved knowledge was also calculated, and statistical significance was considered at *p ≤* 0.05.

**Results:**

There was no significant difference between the participants in the two arms, except for the level of education (*p =* 0.002). The knowledge of the WRA in the intervention arm improved significantly (*p <* 0.001) following the dialogue-based educational intervention by the trained CHVs. None of the demographic characteristics were associated with knowledge.

**Conclusion:**

Dialogue-based educational intervention significantly improved the knowledge of the WRA in the intervention arm, showing its potential to address the knowledge gap in the community.

**Supplementary Information:**

The online version contains supplementary material available at 10.1186/s12905-024-03075-2.

## Background

Cervical cancer continues to be a leading cause of cancer death among women of reproductive age, especially in low- and middle-income countries. In 2020, there were 604,127 reported cases of cervical cancer and 341,831 deaths globally [1]. In Kenya, it is estimated that 5236 women are diagnosed with cervical cancer, with 3211 deaths annually [2]. Cervical cancer thus ranks as the most common cancer among women of reproductive age and the second most common cancer among women of all ages.

Screening, early diagnosis and appropriate treatment have been shown to prevent up to 80% of cervical cancer deaths [3]. However, the uptake of screening in low- and middle-income countries remains dismal, with an estimated 95% of women not having been screened ever. This leads to late diagnosis and poor treatment outcomes, thus significantly increasing the burden of the disease in these regions. This is mainly due to a lack of awareness among the population [3].

In Kenya, screening rates range between 14% and 16% for women aged between 18 and 69. In Kisumu County, facility-based reports from KHIS 2019 [4] indicated that only 5.7% of the eligible women were screened in 2019, and only 29,130 were screened in 2020. It was therefore critical to undertake public health education focused on cervical cancer screening and treatment as part of the activities undertaken by community health volunteers (CHVs) under the community strategy arrangement. We thereafter set out to evaluate the effectiveness of dialogue-based sensitization to enhance cervical knowledge among women of reproductive age in rural Kisumu County.

## Methods

### Study area

**Fig. 1:**
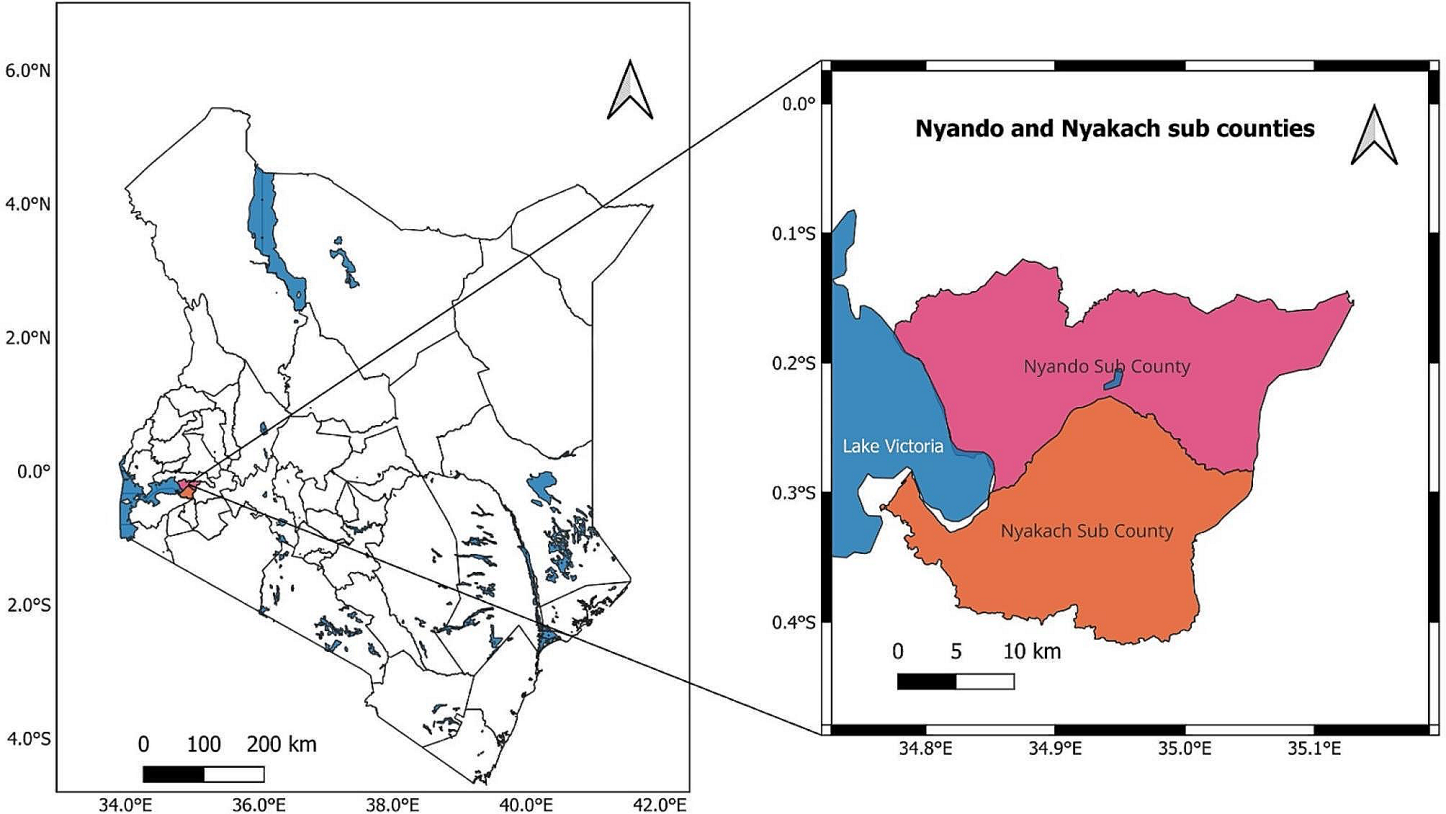
Study location

The study was carried out in the rural sub-counties of Kisumu County in Nyando (Intervention group) and Nyakach sub-counties (Control group) (Fig. 1). According to the 2019 Kenya National Census, the Nyando sub-County covers an area of 413.20 square kilometres with an estimated population of 161,508, 40,468 of which are WRA, while the Nyakach sub-County covers an area of 357.30 square kilometres with an estimated population of 150,320, of which 38,011 are WRA [5].

The two adjacent sub-counties are in the South-Eastern part of Kisumu County, with Nyakach bordering Homabay County to the South, Kericho County to the East and Lake Victoria to the West. The Nyando sub-county border Kericho County to the East, the Kisumu East sub-county and Lake Victoria to the West and the Muhoroni to the North. The major economic activities in both sub-counties are small-scale farming and fishing, with a poverty level of 43% [6].

The two sub-counties were used because of their low cervical cancer screening uptake rates despite having robust and functional community health units and because of their comparability in terms of their rural nature [7]. CHVs are the first level of healthcare provision, linking the community to health facilities and serving as the entry point to the healthcare system, providing basic services such as malaria testing and deworming, among other basic health services.

### Map of Nyando and Nyakach Sub-counties, Kisumu County where the study was conducted

#### Study design

This study adopted a quasi-experimental design (before and after with control group) in which data were collected before (baseline) and after (post) implementation of the intervention with a control group. The intervention was implemented in the health facilities in the Nyando sub-County, and to control for other programmes and interventions that might also have occurred during the study period, the Nyakach sub-County was used as the control site. The cervical cancer screening efforts of the two sub-counties were supported by the Family AIDS and Education Services (FACES) who were the implementing partners for HIV care and treatment in Kisumu County. To minimize the risk of contamination between the two sites, the facilities at the border of the two sub-counties were excluded from the study.

### Target population

The study targeted women of reproductive age (15–49 years) residing in the Nyando and Nyakach sub-counties and the CHVs working in the two sub-counties. According to the 2019 Kenya National Census Report [5], the two sub-counties had a population of 75,364 women of reproductive age.

The sample size for each arm was calculated using the following formula [8]:

$${n_i} = \left\{ {{p_1}\left( {1 - {p_1}} \right) + {p_2}\left( {1 - {p_2}} \right)} \right\}{\left( {\frac{Z}{E}} \right)^2}$$

where n = required sample size for each arm

p1 and p2 are the current cervical cancer screening rates in the county (6% according to KHIS 2019 data).

z = the value from the standard normal distribution at 95% CI (z = 1.96 for 95%).

e = degree of accuracy (0.05).

Therefore:

n={0.06(1-0.06) + 0.06(1-0.06)}(1.96/0.05)^2^.

={0.06(0.94) + 0.06(0.94)}(39.2)^2^.

*n* = 174 participants in each arm + 10% for non-response.

*n* = 192 in each arm.

The sample size was therefore 384 WRA.

The sample of 384 participants was then distributed according to the individual facility’s catchment, based on the number of community units attached to it (Supplementary File 1.docx).

### Data collection

The CHVs’ Community Health Information Systems Household Register (MOH 513) was used to systematically identify potential participants for enrolment. In the control arm, 18 WRA and 15 WRA in the intervention arm were systematically selected per CU to achieve the required sample size. The sampling interval was calculated using the following formula:

Sampling interval per CU = Number of households/Required sample size per CU.

Where a household sampled did not have an eligible respondent or was unwilling to participate in the study, the next household in the list was sampled.

The data was collected using semi-structured questionnaires adopted from the University College London Health Behaviour Research Centre’s cervical cancer awareness measurement tool [9], which was divided into four sections: demographic characteristics, risk factors, signs and symptoms, and availability of screening services. The responses on the risk factors, signs and symptoms, and screening services were used to gauge the WRA’s knowledge. Knowledge was assessed before and after the public health education intervention by trained CHVs.

**Fig. 2:**
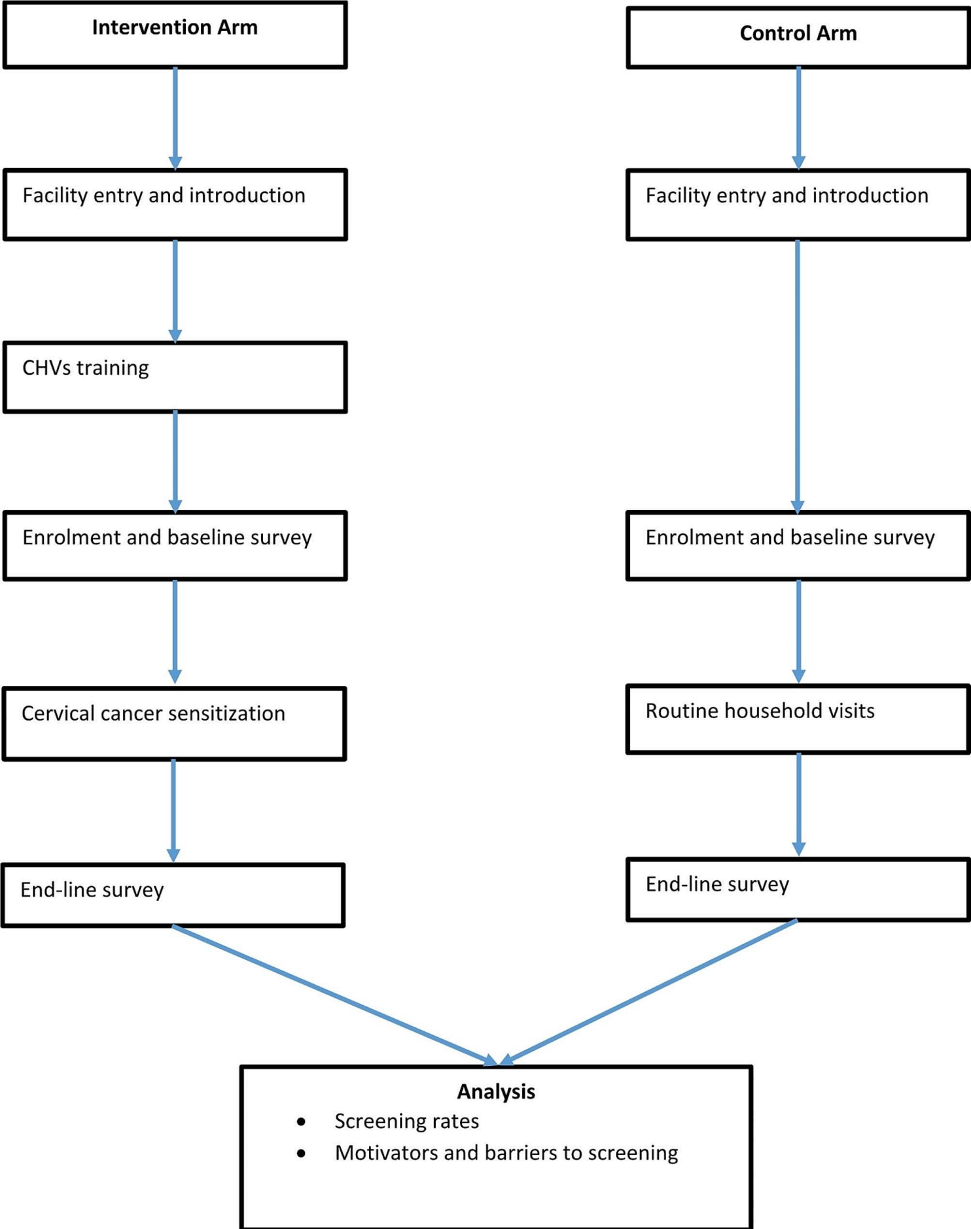
Study flow chart. Flow chart showing the recruitment, intervention and data analysis for the study

In the intervention arm, the CHVs were trained about cervical cancer using the modified CHV training manual (Supplementary File 2.docx). The training was conducted between 20th September and 29th October 2021. The sessions were conducted on a weekly basis, lasted approximately two hours in the morning and consisted of between 10 and 20 CHVs per session, depending on the number of CHVs attached to the facility. Low volume facilities having only one CU and in close proximity had joint sessions to make the 20 CHVs per session. The training was divided into two major parts, with the first part being theory, which had four sessions covering topics on non-communicable diseases with emphasis on cancer, risk factors for cervical cancer, signs and symptoms of cervical cancer, and prevention of cervical cancer through screening and early diagnosis. This session was a guided discussion where each CHV was allowed to share their understanding of the topic with the study staff who provided guidance. The second part involved two practical and role-playing sessions in which each CHV participated in discussions about cervical cancer. Each CHV was required to attend at least three theory and one practical session to be considered adequately trained to participate in the intervention and to be included in the final analysis. After the training sessions, the CHVs were allowed to carry out sensitization in the community among the households in their respective CUs for a period of 10 months between November 2021 and August 2022. As the CHVs visited the households for their routine visits, they also shared information about cervical cancer with the household members through a dialogue-based approach allowing for discussion. At the control site, the traditional health talks and routine home visits normally performed by the CHVs were allowed to continue, with no additional information on cervical cancer (Fig. 2**)**.

### Data analysis

Univariate analysis was performed to understand the demographic characteristics of the study participants. Frequencies, percentages and proportions were determined and presented in tables and graphs. To determine the change in knowledge, the responses were scored, and proportions calculated for each category of knowledge assessment (risk factors, signs and symptoms and service availability). Overall knowledge was calculated as an average score for the knowledge assessment categories, and the percentage scores were calculated. The percentage overall knowledge score was calculated for both the baseline and end-line data. Furthermore, overall knowledge was categorized as poor (less than 0–34%), average (35–74%) or good (75–100%), as proposed previously [10]. The p value was calculated using a test of proportions to determine the statistical significance of the percentage net changes in knowledge score and in the proportion of women with improved knowledge. Net changes in proportion with *p* ≤ 0.05 were considered to indicate statistical significance.

For the demographic characteristics associated with knowledge, a chi-square test was performed to determine the associations between the test scores and the demographic characteristics, with *p* ≤ 0.05 indicating statistical significance. However, in cases where the demographic variables were continuous, we used the Kruskal-Wallis test as the non-parametric test. The non-parametric test was preferred since it does not assume any distribution and hence is not affected by the non-normality of the data.

## Results

The study enrolled 384 participants; however, five could not complete the end-line survey and thus were excluded from the final analysis. Of the five excluded participants, four had moved out of the study area and could not be reached, while one withdrew from the study. Therefore, the final analysis included 189 respondents from the control arm and 190 from the intervention arm.

### Demographic characteristics of the study respondents

There was no significant difference between the two groups except for the level of education (*p =* 0.002) (Table 1).

**Table 1:** Demographic characteristics of the study respondents

Variable	Categories	Intervention (*n* = 190)	Control (*n* = 189)	
		n (%)	n (%)	p-value
Age	18–24	39 (20.5)	32 (16.9)	0.843
	25–30	56 (29.5)	66 (34.9)	
	31–34	43 (22.6)	42 (22.2)	
	35–40	34 (17.9)	29 (15.3)	
	41–44	15 (7.9)	17 (9.0)	
	45–49	3 (1.6)	3 (1.6)	
Education level	No education	1 (0.5)	0 (0.0)	**0.002**
	Primary	56 (29.5)	39 (20.6)	
	Secondary	100 (52.6)	86 (45.5)	
	Tertiary	33 (17.4)	64 (33.9)	
Religion	Christian	188 (98.9)	184 (97.4)	0.326
	Muslim	2 (1.1)	3 (1.6)	
	No religion	0 (0.0)	2 (1.0)	
Marital status	Single	33 (17.4)	35 (18.5)	0.880
	Married	133 (70.0)	133 (70.4)	
	Divorced/widowed/separated	24 (12.6)	21 (11.1)	
Occupation**	Small scale farming	67 (34.5)	56 (28.6)	0.118
	Commercial farming	1 (0.5)	2 (1.0)	
	Business	76 (39.2)	80 (40.8)	
	Formal employment	12 (6.2)	26 (13.3)	
	Casual employment	20 (10.3)	12 (6.1)	
	Other	18 (9.3)	20 (10.2)	
HIV status	Positive	37 (19.5)	37 (19.6)	0.163
	Negative	147 (77.4)	151 (79.9)	
	Don’t know	6 (3.1)	1 (0.5)	

The data are presented as numbers (proportions). **Multiple responses were allowed. The level of significance was determined at *p* ≤ 0.05

### Knowledge of cervical cancer among women of reproductive age in rural Kisumu sub-counties

At baseline, the control arm had significantly better knowledge scores than did the intervention arm (*p <* 0.001). Following dialogue-based community health education by trained community health volunteers, women of reproductive age in the intervention arm had significantly better scores than those in the control arm (*p =* 0.035). The overall knowledge score at the end of the study was also significantly greater than the baseline score (*p =* 0.006).

Further analysis showed that within the arms, there was a significant improvement in the knowledge score for the intervention arm (*p <* 0.001) following dialogue-based community health education by trained CHVs. In the control arm, however, there was a statistically significant decrease in the scores at the end of the study (*p =* 0.002) (Table 2).

**Table 2:** Pre- and post-intervention knowledge on cervical cancer among women of reproductive age in Rural Kisumu County

Baseline vs. End-line						
	Baseline (*n* = 379)			End-line (*n* = 379)		
	Intervention (*n* = 190)		Control (*n* = 189)	Intervention (*n* = 190)		Control (*n* = 189)
**Mean (SD)**	19.4 (11.2)		30.5 (14.0)	28.2 (10.5)		26.0 (12.7)
**Median (IQR)**	16.0 (8.0, 28.0)		28.0 (20.0, 40.0)	28.0 (20.0, 36.0)		24.0 (16.0, 36.0)
**Mean Difference (SE)**	11.1 (0.7)			-2.2 (1.2)		
***p*** **-value (Wilcoxon Rank Sum Test)**	**< 0.001**			**0.035**		
**Intervention vs. Control**						
	**Intervention (** ***n*** ** = 190)**			**Control (** ***n*** ** = 189)**		
	**Baseline**	**End-line**		**Baseline**	**End-line**	
**Mean (SD)**	19.4 (11.2)	28.2 (10.5)		30.5 (14.0)	26.0 (12.7)	
**Median (IQR)**	16 (8, 28)	28 (20, 36)		28 (20, 40)	24 (16, 36)	
**Mean Difference (SE)**	8.8 (1.1)			-4.5 (1.4)		
***p-*** **value (Wilcoxon Rank Sum Test)**	**< 0.001**			**0.002**		

The data are presented as percentages. SD is the standard deviation. IQR is interquartile range. SE is the standard error. Statistical significance is reported at *p* ≤ 0.05

### Categorization of women of reproductive age in rural Kisumu County

Based on the percentage scores, knowledge was categorized as poor (0–35), average (36–75) or good (76–100). The majority of the respondents in both arms (87.4% in the intervention arm and 64.0% in the control arm) had poor knowledge at baseline. However, of the respondents in the control arm, 35.5% had average knowledge in the control arm, while 12.6% had average knowledge in the intervention arm. Following the dialogue-based educational intervention, the percentage of those with poor knowledge in the intervention arm decreased to 72.6%, while the percentage of those with average knowledge increased to 27.4%. In the intervention arm, there was a significant reduction in the number of respondents who scored ‘poor’ and an increase in the number who scored average at the end-line (*p* < 0.001) (Table 3).

**Table 3:** Knowledge categorization of women of reproductive age in Rural Kisumu County

Test Scores	Intervention (*n* = 190)		Control (*n* = 189)	
	Baseline	End-line	Baseline	End-line
0–35	166 (87.4)	138 (72.6)	121 (64.0)	140 (74.1)
**36–75**	24 (12.6)	52 (27.4)	67 (35.5)	49 (25.9)
**> 75**	0	0	1 (0.5)	0
**p value (chi-square)**	**< 0.001**		0.075	

The data are presented as numbers (proportions). Statistical significance is reported at *p ≤* 0.05

### Sociodemographic factors associated with knowledge of cervical cancer among women of reproductive age in rural Kisumu County

The chi-square test was preferred as the best test to determine the association between two categorical variables, that is, the outcome variable, test scores and demographic categorical variables. In cases where the outcome was categorical but the other demographic variables were continuous, we used the Kruskal-Wallis test for categorical outcomes with more than two levels.

None of the demographic characteristics were found to be significantly associated with knowledge of cervical cancer among the WRA (Table 4).

**Table 4:** Demographic characteristics associated with knowledge among WRA in Rural Kisumu County

	Intervention (*n* = 190)				Control (*n* = 189)			
Variables/scores	0–35 (*n* = 166)	36–75 (*n* = 24)	> 75 (*n* = 0)	p-value (Kruskal- Wallis, chi-square)	0–35 (*n* = 121)	36–75 (*n* = 67)	> 75 (*n* = 1)	p-value (Kruskal- Wallis, chi-square)
**Median Age (IQR)**	31 (25,36)	28 (29,34)	-	0.606	27 (31,36)	25 (29,38)	27 (27,27)	0.593
**Education**								
No school	1 (0.6)	0	0	0.227	0	0	0	0.387
Primary	51 (30.7)	5 (20.8)	0		27 (22.3)	12 (17.9)	0	
Secondary	83 (50.0)	17 (70.8)	0		58 (47.9)	28 (41.8)	0	
Tertiary	31 (18.7)	2 (8.3)	0		36 (29.8)	27 (40.3)	1 (100)	
**Religion**								
No religion	0	0	0	0.589	1 (0.8)	1 (1.5)	0	0.755
Christian	164 (98.8)	24 (100)	0		117 (96.7)	66 (98.5)	1 (100)	
Muslim	2 (1.2)	0	0		3 (2.5)	0	0	
**Marital status**								
Single	29 (17.5)	4 (16.7)	0	0.816	21 (16.1)	13 (19.4)	1 (100)	0.334
Married	117 (70.5)	16 (66.7)	0		86 (71.7)	47 (70.2)	0	
Separated	20 (12.1)	4 (16.7)	0		14 (12.3)	7 (10.5)	0	
**Occupation**								
Small scale farming	56 (33.7)	9 (37.5)	0	0.057	32 (26.4)	17 (25.3)	0	0.063
Large scale farming	0	1 (4.2)	0		2 (1.7)	0	0	
Business	68 (41.0)	8 (33.3)	0		47 (38.8)	32 (47.8)	0	
Formal employment	8 (4.8)	3 (12.5)	0		16 (13.2)	11 (16.4)	0	
Casual labour	16 (9.6)	3 (12.5)	0		7 (5.8)	4 (6.0.)	1 (100)	
Housewife	5 (3.0)	0	0		1 (0.8)	0	0	
Student	13 (7.8)	0	0		16 (13.2)	3 (4.5)	0	

The data are presented as percentages. SD is the standard deviation. IQR is interquartile range. SE is the standard error. Statistical significance is reported at *p ≤* 0.05

## Discussion

The results of this study indicate that the majority of the respondents had poor cervical cancer knowledge at baseline; however, following the dialogue-based educational intervention, the knowledge significantly improved in the intervention arm. In the intervention arm, the number of participants who reported poor knowledge decreased, while the number of participants who reported average knowledge increased; however, no participants reported good knowledge even after the intervention. On the other hand, in the control arm, the number of respondents with poor knowledge increased, while the number of those with average and good knowledge decreased at the end of the study.

These results are similar to those of a systematic review of school-based health education, which revealed that educational intervention improved knowledge of cervical cancer and HPV infection [11]. Similarly, findings from another study among parents reported improved knowledge of cervical cancer prevention through HPV vaccination following an educational intervention [12]. Another study evaluating the effect of educational intervention among employed women and female undergraduate students reported improved knowledge after educational intervention [13].

A systematic review to analyse educational interventions aimed at improving the knowledge of middle adolescents on HPV and associated cancers reported improved knowledge and awareness after the interventions [14]. Similarly, a study among women in Lesotho reported a lack of information on cervical cancer screening despite having heard about cervical cancer [15]. However, Vidhya et al. [10]. reported that cancer knowledge among cancer patients was moderately adequate. However, this was among hospital patients who were already receiving treatment, which is normally accompanied by educational sessions and counselling.

The poor knowledge among reproductive-aged women is due to the weakness of the community health education system, which does not place enough emphasis on non-communicable diseases, including cancer. Currently, the use of mainstream media and widely used posters does not seem to effectively reach the target audience. Despite the airing of several advertisements on the need to screen for cervical cancer, posters being pinned on strategic locations in public places, the knowledge of the women targeted with these messages remains low. The dialogue-based approach used in the current study, which also allows for feedback to the health system, has great potential to improve WRA knowledge on cervical cancer and increase the demand for screening services.

Although not significant, there was a notable decrease in the knowledge scores among the participants in the control arm. This could be attributed to the weakness of the current training curriculum to address the misinformation and myths, packaging of the information or mode of delivery. Future studies are needed to interrogate understand the possible causes of this observation.

This study was limited in the duration of follow-up and therefore could not assess knowledge retention over time. It was also limited to only facilities that had reported conducting cervical cancer screening. Finally, as this was a quasi-experimental study, there was potential bias due to differences in the two study sites, and we could not conclusively attribute the improvement in knowledge to the intervention.

## Conclusion

The majority of the WRA respondents had poor knowledge of cervical cancer. This improvement following the dialogue-based public health education intervention indicates that the intervention can be used to improve women’s knowledge of cervical cancer. This model can be emulated in other settings and scaled up to address the cervical cancer knowledge gap.

### Electronic supplementary material

Below is the link to the electronic supplementary material.

Supplementary Material 1

Supplementary Material 2

Supplementary Material 3

## Data Availability

Data is provided within the manuscript or supplementary information files.

## Abbreviations

CHEW: Community Health Extension Worker
CHV: Community Health Volunteer
CU: Community Health Unit
FACES: Family AIDS and Education Services
KHIS: Kenya Health Information System
KNBS: Kenya National Bureau of Statistics
MUERC: Maseno University Ethics Review Committee
NACOSTI: National Commission for Science, Technology & Innovation
NCD: Non-communicable Diseases
WHO: World Health Organization
WRA: Women of Reproductive Age

## References

[1] Stelzle D, Tanaka LF, Lee KK (2021). Estimates of the global burden of cervical cancer associated with HIV. Lancet Glob Heal.

[2] GLOBOCAN. *Kenya: Cancer Fact Sheets*., 2020.

[3] WHO. WHO | Comprehensive cervical cancer control. World Heal Organ 2014. 25642554

[4] KHIS. Cancer Screening Program Monthly Summary Form. 2019.

[5] KNBS. *2019 KENYA POPULATION AND HOUSING CENSUS*. Naiorobi, 2020.

[6] KNBS. *2014 Demographic and Health Survey: Key Indicators*., 2015.

[7] KNBS. *2014 Demographic and Health Survey*., 2015.

[8] Willan AR, Briggs AH. Power and sample size determination. Stat Anal Cost-effectiveness Data 2006:93–116.

[9] UCL. Cervical Cancer Awareness Measure Toolkit Version 2. 2011.

[10] Vidhya K, Gupta S, Lekshmi R et al. Assessment of patient’s knowledge, attitude, and beliefs about cancer: An institute-based study. 2022, 10.4103/jehp.jehp_733_21. 10.4103/jehp.jehp_733_21 PMC8974982 35372615

[11] Ampofo AG, Boyes AW, Khumalo PG (2022). Improving knowledge, attitudes, and uptake of cervical cancer prevention among female students: a systematic review and meta-analysis of school-based health education. Gynecol Oncol.

[12] Sitaresmi MN, Rozanti NM, Simangunsong LB, et al. Improvement of parent’s awareness, knowledge, perception, and acceptability of human papillomavirus vaccination after a structured-educational intervention. BMC Public Health. 2020;20. 10.1186/S12889-020-09962-1. 10.1186/s12889-020-09962-1 PMC7708115 33256697

[13] Chang IJ, Huang R, He W, et al. Effect of an educational intervention on HPV knowledge and vaccine attitudes among urban employed women and female undergraduate students in China: a cross-sectional study. BMC Public Health. 2013;13. 10.1186/1471-2458-13-916. 10.1186/1471-2458-13-916 PMC3852612 24088392

[14] Flood T, Wilson IM, Prue G, et al. Impact of school-based educational interventions in middle adolescent populations (15-17yrs) on human papillomavirus (HPV) vaccination uptake and perceptions/knowledge of HPV and its associated cancers: a systematic review. Prev Med (Baltim). 2020;139. 10.1016/J.YPMED.2020.106168. 10.1016/j.ypmed.2020.106168 32603795

[15] Ramathebane MM, Sooro MA, Kabuya RM, et al. Knowledge and attitudes relating to cervical and breast cancer among women in Maseru, Lesotho. Afr J Prim Heal care Fam Med. 2022;14. 10.4102/PHCFM.V14I1.3459. 10.4102/phcfm.v14i1.3459 PMC9772699 36546486

