# Supplementary material for: Enhancing cervical cancer knowledge among women of reproductive age: a dialogue-based community health education intervention in rural Kisumu County, Kenya
Source: BMC Womens Health. 2024 Jun 6;24:327. doi: 10.1186/s12905-024-03075-2 (PMC11155059; doi:10.1186/s12905-024-03075-2)
Supplement: Supplementary file 1 — Supplementary Material 1 [file 12905_2024_3075_MOESM1_ESM.docx]

MODIFIED COMMUNITY HEALTH VOLUNTEERS TRAINING GUIDE

Training objectives;

1. Explain how to conduct dialogue as a way of communication

2. Explain about cervical cancer

3. Identify the common signs and symptoms of cervical cancers

4. Explain the risk factors for cervical cancers

5. Explain screening options for cervical cancer

Guidelines for Conducting Dialogue

1. Be sensitive to the people’s experiences
2. Respect for differences and mutual trust
3. Use participatory approach with space for listening, inclusion and expression of concerns
4. Facilitate the process of self-development and reflection
5. Encourage mutual learning among the participants
6. Ask the what, why, who, when and how questions when analyzing the problem.

Steps in Community Dialogue

**Step 7**

Evaluate together and provide feedback

**Step 1**

Problem Identification

**Step 6**

Acting together

**Step 2**

Problem analysis

**Step 3**

Identification of best option(s)

**Step 5**

Planning together

**Step 4**

Prioritization of options

Prioritization of options

The Role of the Community Health Volunteer (CHV) in NCD Prevention and Control

Discuss with community members on the importance of regular screening for NCDs such as diabetes, hypertension and cancers.

Educate community members on the signs and symptoms of common NCDs

Provide information on prevention of complications once diagnosis is made

Provide health information on health lifestyle including healthy eating, exercising and not smoking as a way of lowering the risk of NCDs.

Conduct screening for diabetes and hypertension within the community in consultation with the CHA

Facilitate referral of community members suspected of having NCDs to the nearest health facility for appropriate management and follow-up.

Assist the health worker in the follow-up of persons being managed for NCDs including tracing those who have defaulted on treatment.

Support treatment adherence among community members on management for NCDs.

Provide psycho-social support to community members being managed for NCDs by encouraging them to form support groups.

Maintain an updated record of NCD-related health activities conducted within the Community

Cancer situation in Kenya

Cancer is one of the leading causes of death in Kenya.

Common types of cancer seen in Kenya are cancers of the cervix, breast, esophagus and prostate.

Others include head and neck, colon and rectum, stomach, liver among others

HIV associated cancers are also on the increase and affect various regions of the body.

Terms like swelling, tumor, *uvimbe*, *saratani*, and growth are commonly used to refer to cancer

Causes and risk factors

Genetics

Tobacco use

Unhealthy diet

Physical inactivity

Sun and ultra-violet rays’ exposure

Having many children

Having many sexual partners

Early onset of sexual activity

HPV infection

Smoking

Immune suppression

Signs and symptoms of cervical cancer

Abnormal vaginal bleeding

Abnormal vaginal discharge

Abdominal pains

Pain during sex

Bleeding after intercourse

Bleeding after menopause

Bleeding between periods

Bleeding after douching

Bleeding following a pelvic exam

Having heavier menstrual periods than usual or ones that last longer than usual

Pelvic pain

Cervical cancer screening services

Methods

VIA

VILI

Pap-smear

HPV testing

Duration of the procedure: less than 30 minutes

Cost: free in government health facilities

Rescreening:

Once every 5 years except for HIV-infected women and those with abnormal tests. If screening has been normal, it can be stopped at 65 years of age. If results have been abnormal or the client has undergone treatment, rescreening should be provided in a year.

Annual screening is not recommended for the HIV negative population. Screening for HIV-infected Women

All HIV-positive women aged 18 years and above should be screened for cervical cancer. Screening should begin at diagnosis of HIV, 6 monthly in the first year, then yearly thereafter. Screening during Pregnancy and Puerperium

Screening during pregnancy until 20 weeks gestation is recommended.

No treatment should be given for premalignant lesions during pregnancy, unless there is evidence of a malignant lesion.

Patients should return at 6-12 weeks post-partum for treatment. Since most women also bring their children for immunization at 6 weeks post-partum, eligible National Guidelines for Cancer Management Kenya 87 women will be offered screening at that time and managed accordingly.

Screening done by VIA or VILI. 1. Negative cases: wait for 5 years for another screening. 2. Positive cases: Those suitable for cryotherapy should be offered the procedure. Those not suitable should be referred for colposcopy and biopsy. 3. Those found suspicious at screening should also be referred for colposcopy and biopsy

Women who have had their uterus and cervix removed in a hysterectomy and have no history of cervical cancer or pre-cancer should not be screened

Women who have had the HPV vaccine should still follow the screening recommendations for their age group
