# Supplementary material for: Enhancing cervical cancer knowledge among women of reproductive age: a dialogue-based community health education intervention in rural Kisumu County, Kenya
Source: BMC Womens Health. 2024 Jun 6;24:327. doi: 10.1186/s12905-024-03075-2 (PMC11155059; doi:10.1186/s12905-024-03075-2)
Supplement: Supplementary file 3 — Supplementary Material 3 [file 12905_2024_3075_MOESM3_ESM.docx]

**Distribution of Study Participants per Participating Facilities**

| S/No. | Health Facility | Sub-County | Arm | Number of CU. | Respondents |
| --- | --- | --- | --- | --- | --- |
| 1 | Bonde | Nyakach | Control | 1 | 18 |
| 2 | Pedo | Nyakach | Control | 1 | 18 |
| 3 | Onyuongo | Nyakach | Control | 1 | 18 |
| 4 | Radienya | Nyakach | Control | 1 | 18 |
| 5 | Sigoti | Nyakach | Control | 2 | 34 |
| 6 | Oboch | Nyakach | Control | 1 | 18 |
| 7 | Kibogo | Nyakach | Control | 1 | 18 |
| 8 | Kodingo | Nyakach | Control | 3 | 50 |
| 9 | Bunde | Nyando | Intervention | 1 | 15 |
| 10 | Magina | Nyando | Intervention | 1 | 15 |
| 11 | Koduol | Nyando | Intervention | 1 | 15 |
| 12 | Nyangande | Nyando | Intervention | 3 | 44 |
| 13 | HongoOgosa | Nyando | Intervention | 2 | 29 |
| 14 | Kochieng | Nyando | Intervention | 1 | 15 |
| 15 | Okana | Nyando | Intervention | 1 | 15 |
| 16 | Kadinda | Nyando | Intervention | 1 | 15 |
| 17 | Nyakongo | Nyando | Intervention | 2 | 29 |
|  | Total | NA |  | 24 | 384 |

The distribution of the study participants was done proportionally based on the number of community units attached to each facility. Decimals rounded up to the nearest whole number.
